# Current Concepts in the Treatment of Giant Cell Tumors of Bone

**DOI:** 10.3390/cancers13153647

**Published:** 2021-07-21

**Authors:** Shinji Tsukamoto, Andreas F. Mavrogenis, Akira Kido, Costantino Errani

**Affiliations:** 1 Department of Orthopaedic Surgery, Nara Medical University, 840, Shijo-cho, Kashihara City 634-8521, Nara, Japan; 2 First Department of Orthopaedics, School of Medicine, National and Kapodistrian University of Athens, 41 Ventouri Street, Holargos, 15562 Athens, Greece; afm@otenet.gr; 3 Department of Rehabilitation Medicine, Nara Medical University, 840, Shijo-cho, Kashihara City 634-8521, Nara, Japan; akirakid@naramed-u.ac.jp; 4 Department of Orthopaedic Oncology, IRCCS Istituto Ortopedico Rizzoli, Via Pupilli 1, 40136 Bologna, Italy; costantino.errani@ior.it

**Keywords:** giant cell tumor of bone, denosumab, surgery, metastasis, malignant transformation, bisphosphonate, recurrence

## Abstract

**Simple Summary:**

According to the 2020 World Health Organization classification, a giant cell tumor of bone is an intermediate malignant bone tumor. Denosumab treatment before curettage should be avoided due to the increased risk of local recurrence. Administration of denosumab before en bloc resection of the giant cell tumors of the pelvis and spine facilitates en bloc resection. Nerve-sparing surgery after embolization is a possible treatment for giant cell tumors of the sacrum. Denosumab therapy with or without embolization is indicated for inoperable giant cell tumors of the pelvis, spine, and sacrum. A wait-and-see approach is recommended for lung metastases at first, then denosumab should be administered to the growing lesions. Radiotherapy is not recommended owing to the risk of malignant transformation. Local recurrence after 2 years or more should be indicative of malignant transformation. This review summarizes the treatment approaches for non-malignant and malignant giant cell tumors of bone.

**Abstract:**

The 2020 World Health Organization classification defined giant cell tumors of bone (GCTBs) as intermediate malignant tumors. Since the mutated H3F3A was found to be a specific marker for GCTB, it has become very useful in diagnosing GCTB. Curettage is the most common treatment for GCTBs. Preoperative administration of denosumab makes curettage difficult and increases the risk of local recurrence. Curettage is recommended to achieve good functional outcomes, even for local recurrence. For pathological fractures, joints should be preserved as much as possible and curettage should be attempted. Preoperative administration of denosumab for pelvic and spinal GCTBs reduces extraosseous lesions, hardens the tumor, and facilitates en bloc resection. Nerve-sparing surgery after embolization is a possible treatment for sacral GCTBS. Denosumab therapy with or without embolization is indicated for inoperable pelvic, spinal, and sacral GCTBs. It is recommended to first observe lung metastases, then administer denosumab for growing lesions. Radiotherapy is associated with a risk of malignant transformation and should be limited to cases where surgery is impossible and denosumab, zoledronic acid, or embolization is not available. Local recurrence after 2 years or more should be indicative of malignant transformation. This review summarizes the treatment approaches for non-malignant and malignant GCTBs.

## 1. Introduction

**Figure 1:**
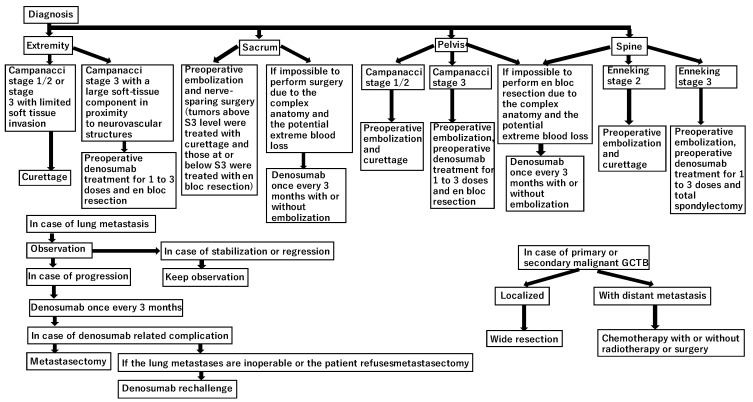
Treatment algorithm for giant cell tumors of the bone. GCTB: giant cell tumor of bone.

Giant cell tumors of bone (GCTBs) are intermediate malignant bone tumors with high local infiltration ability, which accounts for approximately 5% of all primary bone tumors [1]. The predominant age is approximately 30 years [1]. Histologically, a GCTB is composed of neoplastic mononuclear stromal cells with a monotonous appearance mixed with macrophages and osteoclast-like giant cells [1]. Since the discovery of the H3.3 p.Gly34Trp immunohistochemistry as a marker specific to GCTBs, it has been used for the diagnosis of GCTB [2,3,4]. Approximately 1–9% of patients with GCTBs develop lung metastases [5,6,7,8,9] and 2.4% develop secondary malignant GCTBs [10]. GCTBs most frequently occur in the distal femur (30%), proximal tibia (28%), distal radius (9%), and distal tibia (6%) [11]. Pelvic (2%), sacral (2%), and spinal (3%) GCTBs are extremely rare [11]. To date, no medical treatment has been approved for GCTBs; however, denosumab (a fully human monoclonal antibody that inhibits the receptor activation of nuclear factor-kappa β ligand) was approved by the United States Food and Drug Administration in 2013 owing to its efficacy and safety in the treatment of GCTBs [12]. Moreover, denosumab has a downstaging effect to less invasive surgery [13]. Currently, denosumab treatment is indicated for GCTBs that are inoperable or cause severe dysfunction even after resection [12]; however, denosumab administration before curettage increases the local recurrence rate [14,15,16,17,18,19], and some concerns have been raised regarding its association with malignant transformation [12,13,16,20,21,22,23,24,25,26,27]. In addition, because complications have been reported, such as osteonecrosis of the jaw, peripheral neuropathy, skin rash, hypophosphatemia, and atypical femoral fracture associated with long-term administration of denosumab [28], a clinical trial on denosumab treatment (120 mg every 3 months) using a reduced dose density to decrease these complications in patients with unresectable GCTB (REDUCE study) (NCT03620149) is currently ongoing. In this review article, we summarize the treatment approaches for non-malignant and malignant GCTBs (Figure 1).

## 2. Giant Cell Tumor of the Extremities

For Campanacci stage 1 and stage 2 GCTBs, curettage should be the standard treatment because it can preserve joints and is associated with good functional outcomes [11,29]. Aggressive curettage with a high-speed burr is recommended [29,30]. In addition, adjuvants such as phenol, ethanol, liquid nitrogen, and electrocautery have been used as local adjuvant treatments, although there is no evidence indicating the superiority of one local adjuvant treatment over another [30]. The use of a high-speed burr and local adjuvant therapy has been reported to reduce local recurrence rates [30]. It is also recommended to curette the tumor through a large cortical window to reduce blind spots during curettage [29]. Polymethylmethacrylate (PMMA), bone allografts, and hydroxyapatite have been used to fill the defects. The advantages of PMMA are that it has an antitumor effect due to the application of heat, local recurrence can be easily detected by imaging, and early weight bearing is possible [29]; however, when PMMA is used to fill the subchondral bone, cartilage damage can possibly occur because of the heat produced during polymerization, thereby increasing the risk of mechanical failure [31,32]. Mechanical failures such as osteoarthritis progression and periarticular fractures have been reported to occur in 10% (2 of 21) of cases in the PMMA and periarticular bone graft group and 55% (12 of 22) of cases in the PMMA only group [31]; therefore, subchondral lesions should be filled with bone allografts and PMMA [31]. A recent systematic review reported that the use of one or two high-speed burrs, PMMA, or phenol could reduce the local recurrence rate by 50% compared to the use of simple curettage [33]. The use of a high-speed burr, PMMA, and phenol resulted in a local recurrence rate of 11% [33]. Furthermore, in a study by Balke et al., the recurrence rate after re-curettage for recurrent lesions was 22% [34]; therefore, curettage seems to be a treatment option for local recurrence to achieve good functional outcomes [30].

The efficacy of zoledronic acid in the treatment of GCTBs remains controversial. Various in vitro and in vivo studies have shown that zoledronic acid inhibits neoplastic stromal cells, apoptosis, and osteogenic differentiation [35,36,37,38,39,40]. In 2008, Tse et al. conducted a retrospective study of preoperative and postoperative bisphosphonate therapy for GCTBs in the extremities and reported that one of 24 (4.2%) patients who underwent preoperative and postoperative bisphosphonate therapy and surgery (curettage or en bloc resection) experienced local recurrence and that six of 20 (30%) patients who underwent only surgery experienced local recurrence [41]. They reported that bisphosphonate therapy may reduce postoperative recurrence rates compared with surgery alone [41]. Yu et al. performed curettage and postoperative bisphosphonate therapy in 16 patients with GCTB in the distal femur who were followed-up for a median of 25 months and reported the absence of recurrence [42]. Gouin et al. administered five courses of zoledronic acid (4 mg intravenously [IV] every 3 weeks) after curettage in 24 patients with GCTB and reported that local recurrence was observed in four (15%) patients [43]. Lipplaa et al. conducted a multicenter randomized phase II trial comparing the local recurrence rate after surgery between the adjuvant zoledronic acid group (*n* = 8; 4 mg IV at 1, 2, 3, 6, 9, 12 months postoperatively) and the placebo group (*n* = 6) [44]. During a median follow-up of 94 months, the recurrence rates were 38% (3 of 8 patients) in the adjuvant zoledronic acid group and 17% (1 of 6 patients) in the placebo group (*p* = 0.58) [44], indicating that zoledronic acid was not effective. In contrast, Dubey et al. conducted another randomized trial of neoadjuvant zoledronic acid combined with surgery (*n* = 15) versus surgery alone (*n* = 15) in patients with GCTB of the extremities [45]. In the neoadjuvant zoledronic acid combined with surgery group, neoadjuvant zoledronic acid treatment (5 mg IV was administered three times every 4 weeks) was followed by surgery in 13 of 15 patients (curettage, 12 patients; resection, 1 patient; no surgery, 2 patients), while in the surgery only group, 13 patients underwent curettage and two patients underwent en bloc resection [45]. Pain was reduced (visual analog scale score, 5.3–1.8) and follow-up radiography showed increased mineralization at the periphery of the lesion in the neoadjuvant zoledronic acid combined with surgery group [45]. Bisphosphonate treatment succeeded in controlling tumor growth, as no growth was observed 3 months after administration of neoadjuvant zoledronic acid [45]. In addition, the tumor cells had a significantly higher apoptosis index after administration of zoledronic acid (mean 41% in the neoadjuvant zoledronic acid combined with surgery group versus a mean of 6% in the control group) [45]. 

A few studies have reported that the use of PMMA mixed with bisphosphonates reduces the local recurrence rate after curettage. Zwolak et al. studied the elution dynamics of zoledronic acid release from PMMA and its antitumor effects on GCTBs in vitro [46]. They found that zoledronic acid remains biologically active despite the polymerization of cement [46]. Its release was the highest in the first 24 h at various concentrations and reached a plateau after 4 days [46]. Zoledronic acid showed a high cytotoxic effect on GCTB stromal cells and a dose-dependent decrease in the number of surviving cells was observed [46]. Chen et al. [47] filled cavities with PMMA mixed with bisphosphonates after curettage in four patients with sacral GCTB. The radiographs showed increased sclerosis surrounding the PMMA after a median follow-up of 28 months [47]. Greenberg et al. filled cavities with PMMA mixed with bisphosphonates after curettage in 17 patients [48]. Local recurrence was observed in one patient (6%) after an average follow-up of 52 months [48]. No localized or systemic adverse events were reported [48].

Numerous studies have reported that the administration of denosumab before curettage increases the local recurrence rate [14,15,16,17,18,19]. A recent systematic review reported that the recurrence rate was 20–100% in a group that received preoperative denosumab therapy and underwent curettage and that it was 0–50% in a group that underwent curettage alone [15]. This may be because preoperative administration of denosumab causes osteosclerosis, which makes it difficult to identify the tumor area intraoperatively, leaving the tumor behind, while the tumor cells hidden in the osteosclerotic lesion are reactivated after denosumab treatment is discontinued [14,15,49,50]. Furthermore, denosumab does not induce apoptosis in GCTB cells [35,51]; however, all previous studies were retrospective in nature and biased, as denosumab was administered to patients with a more aggressive GCTB, meaning no causation could be demonstrated [15]. A randomized control trial comparing the group receiving denosumab for 2 months before curettage and the group that did not receive denosumab by the Japan Clinical Oncology Group (JCOG 1610 study) is currently ongoing [52].

Curettage has been associated with better postoperative limb function than en bloc resection (median Musculoskeletal Tumor Society (MSTS) scores of 29.5 and 27 in the curettage and en bloc resection groups, respectively (*p* = 0.029)) [53]. Limb function after en bloc resection of the fibula and distal ulna is usually good; therefore, Campanacci stage 1 or 2 GCTB in the fibula or distal ulna is an indication for en bloc resection [29]. Pathological fractures were found in 9–17% of patients with GCTB [11,19], while the recurrence rate were similar between curettage and en bloc resection (1/13 patients (7%) and 2/33 patients (6%), respectively) [53]; therefore, pathological fracture is not a contraindication for curettage [53,54,55]. For simple fractures, curettage and plate fixation can be performed; cast fixation is indicated to heal the fracture, followed by curettage. If the fracture is complicated and cannot be fixed with a plate, en bloc resection and reconstruction with a prosthesis or allografts are usually indicated [53,56].

The recurrence rates after curettage seem to be higher in patients with GCTBs in the distal radius, proximal femur, and hand or foot compared to other sites of the limbs; hence, extreme caution is required [29,57,58,59]. The reason for the high recurrence rate of GCTB in the distal radius is that the bone quality of the distal radius is relatively fragile and the distance to the carpal and ulna is very close, making it difficult to perform aggressive curettage [60,61]. The reason for the high recurrence rate of GCTB in the proximal femur is that curettage alone may be inadequate due to the risk of head necrosis and fracture [29]. The reason for the high recurrence rate of GCTB in the hand and foot is that the cortical window needs to be small as the bones in this location are smaller, making it difficult to perform aggressive curettage [59]. The presence of a fluid–fluid interface suggests a secondary aneurysmal bone cyst, while the presence of a secondary aneurysmal bone cyst increases the local recurrence rate after curettage [62,63]. Tang et al. reported that secondary aneurysmal bone cysts were found in 60 (23%) of 256 patients with GCTB in the extremities; the local recurrence rate was 53% in the group with secondary aneurysmal bone cysts, while it was 26% in the group without secondary aneurysmal bone cysts (*p* < 0.05) [62]. The reason for the higher risk of local recurrence is that secondary aneurysmal bone cysts cause more bleeding and inadequate curettage [62]. A higher neutrophil-to-lymphocyte ratio (cutoff value: 2.32) was associated with a higher local recurrence rate (hazard ratio (HR) = 4.18, 95% confidence interval (CI): 1.83–9.57, *p* = 0.001) [64], whereas a higher prognostic nutritional index (PNI) (PNI = albumin (g/L) + 5 × total lymphocyte counts per liter; cutoff value: 48.6) was associated with a lower local recurrence rate (HR = 0.406, 95% CI: 0.222–0.743, *p* = 0.003) [65].

En bloc resection is usually indicated for Campanacci stage 3 GCTB with bone destruction and soft tissue involvement [29]. Treatment guidelines based on the GCTB score around the knee were established in accordance with the Chinese Expert Consensus [66]. As shown in Table 1, the guidelines comprised the following four items: pathological fracture, cortical bone destruction, tumor size, and articular surface involvement. The total score range was 0–12 points; patients with a score of 1–4 points were recommended for curettage, those with a score of 5–9 points were recommended for curettage combined with internal fixation, and those with a score of 10–12 points were recommended for en bloc resection and reconstruction with a prosthesis or allograft (Table 2) [66].

Denosumab administration before en bloc resection may harden the tumor, reduce tumor spillage, and reduce the local recurrence rate; therefore, denosumab administration before en bloc resection should be recommended to facilitate surgery [19,67,68]. Medellin et al. reported a local recurrence rate of 24% (11 of 45 patients) in the en bloc resection alone group and 0% (0 of 3 patients) in the en bloc resection combined with preoperative denosumab therapy group [19]. Sahito et al. reported a local recurrence rate of 4.9% (2 of 41 patients) in the en bloc resection alone group and 3.5% (1 of 29 patients) in the en bloc resection combined with preoperative denosumab therapy group [69]. In particular, extraosseous lesions decreased after denosumab therapy [70]. Preoperative denosumab administration (1–3 doses) is recommended; the local recurrence rates were similar between patients receiving 1–3 doses and those receiving ≥3 doses of denosumab preoperatively (27% (13 of 48 patients) vs. 36% (13 of 36 patients)), reducing the costs and the incidence of denosumab-related complications [59].

## 3. Giant Cell Tumor of the Pelvis

Due to the complex anatomy of the pelvic area and the high local infiltration capacity of GCTBs, there is no standard treatment procedure for pelvic GCTB. Treatment options include curettage [71,72,73,74,75,76], en bloc resection [71,72,74,75,76], and radiotherapy [77,78]. Radiotherapy can cause local damage such as early- and late-onset dermatitis, late-onset pathological fractures, and neuritis [74,77,78,79]. In addition, post-irradiation malignant transformation rates range from 11% to 25% in patients with GCTB of the pelvis [74,76]. Curettage maintained the pelvic integrity, although the local recurrence rates in patients with GCTB in the pelvis ranged from 6.3% to 43% [71,72,74,75,76]. En bloc resection can reduce the local recurrence rate [71,72,74,75,76], although it may increase the risk of complications such as infection, hematoma, and functional loss [80,81,82,83]. Guo et al. [84] retrospectively investigated 27 patients with acetabular GCTB who underwent surgery. Four of 13 (31%) patients who underwent curettage experienced local recurrence, while 0 of 14 (0%) patients who underwent en bloc resection experienced local recurrence. The mean MSTS scores were 24 in 13 patients who underwent curettage and 22 of 14 patients who underwent en bloc resection. Thrombosis (1 patient, 8%) and infection (1 patient, 8%) occurred in two patients who underwent curettage, while infection (5 patients, 36%), non-union (1 patient, 7%), and dislocation (1 patient, 7%) occurred in seven patients who underwent en bloc resection. En bloc resection may be a reasonable option for patients with acetabular GCTB with a low local recurrence rate, although it has a high incidence of complications [84]; thus, if en bloc resection is planned, short-term denosumab treatment should be recommended prior to surgery to reduce the risk of intraoperative bleeding and the local recurrence rate.

## 4. Giant Cell Tumor of the Sacrum

Sacral GCTB has a higher postoperative recurrence rate than pelvic GCTB [74]. Most patients with sacral GCTB have lesions at the S3 level or higher [85]. Complete or partial sacral resection often requires sacrifice of the sacral nerve root and is associated with potentially serious disability [86,87,88]. Todd et al. reported that in patients who underwent bilateral S4–S5 resection with at least one S3 nerve root preserved, normal bowel function was maintained in all patients, while normal bladder function was maintained in 69% of patients [89]. In patients who underwent asymmetric sacral resection with preservation of at least one S3 nerve root, normal bowel function was maintained in 67% of patients, while normal bladder function was maintained in 60% of patients [89]. Huang et al. reported that patients with preserved bilateral S3 nerve roots had significantly lower incidence rates and degrees of urinary incontinence (*p* = 0.003) and abnormal bladder sensation (*p* = 0.039) than those who underwent unilateral S3 nerve root excision [90]; therefore, preservation of the bilateral S3 nerve roots is required to achieve normal bowel and bladder function [90]. Several studies have reported that nerve-sparing surgery for the treatment of sacral GCTB can provide satisfactory local control [91,92]. This method involves curettage of tumors in the cephalad part (above the S3 level) to maintain the function of the sacral nerves, especially S1, S2, and S3 nerves, as well as en bloc resection of tumors in the caudal part (at or below the S3 level) [93]. The caudal nerves (S4 and S5) surrounded by the tumor can be resected, although the cranial nerves (S1–S3) need to be preserved [93]. Zhao et al. reported a local recurrence rate of 29% (33 of 114 patients) in patients with sacral GCTB who underwent nerve-sparing surgery [93]. Multivariate Cox regression analysis showed that large tumor size (>8.80 cm) (HR = 3.16; *p* = 0.014), high neutrophil-to-lymphocyte ratio (>2.09) (HR = 3.13; *p* = 0.012), involvement of the sacroiliac joint (HR = 3.09; *p* = 0.039), and massive intraoperative blood loss (>1550 mL) (HR = 2.47; *p* = 0.022) were independent risk factors for local recurrence [93]. Spinopelvic stabilization should be indicated in patients with an unstable sacroiliac joint after surgical resection of the sacral GCTB [85]. Managing massive bleeding associated with curettage of sacral GCTBs is a major challenge for orthopedic surgeons [86]. Tang et al. reported that tumors cephalad to the S2–S3 disc space (odds ratio (OR), 3.84), tumor volume > 200 cm^3^ (OR, 3.38), and excessive blood supply (OR, 2.28) independently predicted a large amount of blood loss [94]. Aortic balloon occlusion [95] and selective arterial embolization [96] were effective in reducing bleeding. Preoperative denosumab treatment reduced bleeding during curettage and was associated with a shorter surgical time [18,96]; however, osteosclerosis due to denosumab use may make it difficult to perform tumor curettage and could lead to a higher recurrence rate after treatment discontinuation [18,96]. Yang et al. reported that the recurrence rate was higher in the nerve-sparing surgery with the preoperative denosumab therapy group than in the nerve-sparing surgery alone group (67% (4 of 6 patients) vs. 0% (none of 10 patients)) [18]; however, Xu et al. reported that the local recurrence rate in the nerve-sparing surgery with bisphosphonate treatment group (preoperative and 2 years after surgery) was lower than that in the nerve-sparing surgery alone group (11% (2 of 19 patients) vs. 44% (7 of 16 patients)) [97]. 

**Figure 2:**
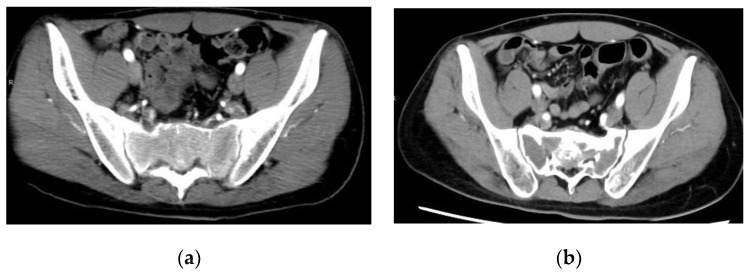
A case of sacral giant cell tumor of the bone treated with denosumab and embolization: (**a**) contrast-enhanced computed tomography at presentation showing osteolytic lesions of the sacrum and contrast-enhanced effects; (**b**) contrast-enhanced computed tomography showing bone sclerosis and loss of contrast effect 16 months after diagnosis, after 16 doses of denosumab and two sessions of embolization. The pain observed at presentation also improved. The patient experienced no complications related to denosumab therapy or embolization.

Persistent neurological dysfunction after surgery for sacral GCTB is a major limiting factor, and patients tend to prefer non-surgical options as treatment for diseases such as GCTB, which are less life-threatening even if they progress [98]. In a retrospective study of 30 patients with sacral GCTB, Chen et al. found that preoperative denosumab treatment reduced pain and neurological deficits in 80% of patients [99]. Tartrate-resistant acid phosphatase 5b expression is a useful tumor marker during denosumab treatment [100,101]. Lin et al. reported the prognosis of 18 patients with sacral GCTB who underwent embolization alone [102]. Kaplan–Meier analysis showed that the risk of local recurrence was 31% at 10 years and 43% at 20 years [102]. According to a systematic review of embolization for sacral GCTB, embolization was performed 1–10 times (mean: 4.1 times) during a mean follow-up period of 85.8 months (range, 2–277 months) [103]. The response rate based on objective imaging was 82% (36 of 44 patients) [103]. The significant changes observed due to embolization were pain relief, diminished vascular distribution on imaging, and ossification around the lesion [85,104]. The interval of embolization was every 4–6 weeks [104]. Puri et al. reported the outcomes of 13 patients with sacral GCTB who underwent non-surgical treatment [98]. The non-surgical treatments included various combinations of short-term denosumab, embolization, and radiotherapy [98]. No further treatment was performed if lesion growth was under control [98]. In cases of lesion growth, additional denosumab treatment or embolization was performed until the tumor was locally controlled [98]. The results showed that 10 (77%) patients experienced non-progressive and asymptomatic disease, two (15%) patients experienced stable disease but experienced occasional pain, and one (8%) patient died due to a different disease [98]. The total numbers of embolizations performed were in the range of 0–12 (mean = 4), while the total numbers of denosumab doses administered were in the range of 5–16 (mean = 9) [98]. Eight patients received radiotherapy. Patients with bladder dysfunction recovered at presentation [98]; therefore, denosumab and embolization are possible treatment options for patients with sacral GCTB who are inoperable or who develop severe neurological dysfunction after surgery (Figure 2).

## 5. Giant Cell Tumor of the Mobile Spine

GCTB of the spine usually occurs in the vertebral body [105]. Surgical methods for spinal GCTB are usually determined based on the Enneking stage [106]. According to the Enneking staging system, most GCTBs are active (S2), defined as symptomatic and remaining intracompartmental, or aggressive (S3), described as symptomatic and extending beyond the compartment. Curettage is indicated for patients with S2 lesions, while piecemeal total spondylectomy or total en bloc spondylectomy is indicated for patients with S3 lesions [106]. Boriani et al. retrospectively investigated 49 patients with spinal GCTB who underwent surgery. Among the 18 patients with Enneking stage 2 tumors, one (6%) of 16 patients who underwent curettage and none of the two patients who underwent total spondylectomy experienced local recurrence. Among the 24 patients with Enneking stage 3 tumors, eight (62%) of 13 patients who underwent curettage and one (9%) of 11 patients who underwent total spondylectomy experienced local recurrence [107]. In the case of GCTB in the cervical spine, the recurrence rate after piecemeal total spondylectomy was 8% (1 of 13 patients) [108]. Yokogawa et al. reported that the recurrence rate after total en bloc spondylectomy with intralesional pediculotomy for Enneking stage 3 spinal GCTB was 0% (0 of 12 patients), while that after piecemeal total spondylectomy for Enneking stage 3 spinal GCTB was 17% (2 of 12 patients) [109]. Boriani et al. reported that no recurrence was observed in four patients with spinal GCTB who received preoperative denosumab treatment and underwent total en bloc spondylectomy [68]; thus, curettage can be indicated for patients with Enneking stage 2 tumors, while total spondylectomy with preoperative denosumab therapy should be indicated for patients with Enneking stage 3 tumors [68]. Xu et al. conducted a retrospective study of 102 patients with spinal GCTB and reported that adjuvant bisphosphonate therapy (preoperative and 2 years postoperatively) significantly reduced the local recurrence rate based on multivariate analysis [110].

GCTBs are highly radiosensitive [111]. According to a systematic review on radiotherapy for spinal GCTB, radiotherapy had a response rate of 100%, overall survival of 98%, and an overall local control rate of 79% [111]. No association was found between the radiation dose and prognosis [111]; however, Chen et al. [112] reported that 33% of patients with GCTBs in any bony location who received radiotherapy experienced malignant transformation. As such, van der Heijden et al. [113] stated that radiotherapy should be restricted in patients with unresectable or recurrent GCTBs (e.g., those in the spine or sacrum) when surgical morbidity is unacceptable and when denosumab is contraindicated or unavailable.

## 6. Lung Metastasis

Lung metastases occur in 1–9% of patients with GCTBs [5,6,7,8,9]. Distal radius [8,9], Campanacci stage 3 [114], and repeated local recurrences [114,115,116,117,118,119] have been associated with lung metastases. A recent study including patients with GCTB of the extremities reported similar lung metastasis rates between the group undergoing surgery and preoperative and postoperative denosumab therapy and the group undergoing surgery alone (3.3% (1 of 30 patients) vs. 4.7% (18 of 381 patients)) [115]. This study suggests that denosumab has no preventive effect on lung metastases [115]. According to a systematic review involving 242 patients with lung metastases from GCTBs, spontaneous regression was observed in 4.5% of patients [120]. Another study reported that 45% (10 of 22) of patients with lung metastases who were initially managed with the wait-and-see approach had stable disease [121]. As such, it is recommended to adopt the wait-and-see approach for lung metastases prior to the selection of treatments [116,117,121,122]; however, nodules measuring ≥ 5 mm have a high risk of growth, and caution is required when treating such nodules [121]. Denosumab should be administered every 3 months for the progression of lung metastases [121]. Palmerini et al. reported a series of 15 patients with metastatic GCTB treated with denosumab and reported that all patients achieved tumor control [28]. Engellau et al. reported that all 38 patients with metastatic GCTB achieved tumor control after receiving denosumab treatment [70]; thus, denosumab can inhibit the progression of almost all lung metastases [28,70]. Metastasectomy is recommended if denosumab-related complications occur [121]. Denosumab re-challenge should be performed in patients with inoperable lesions or in those who refuse surgery [121,123,124]. Balke et al. reported that all 12 patients who had metastatic GCTB and were treated with bisphosphonate experienced stable disease [125].

## 7. Denosumab Alone Therapy

Denosumab therapy may be an alternative to surgery if patients cannot tolerate en bloc resection or if the loss of function after surgery is unacceptable. Palmerini et al. administered denosumab alone to 54 patients with metastatic or unresectable GCTB and reported that 40% (4 of 10) of patients experienced tumor regrowth after 8 months during a median follow-up period of 15 months after denosumab discontinuation [28]; however, some authors have reported that resumption of denosumab treatment is possible and effective [121,123,124] and that bone formation and tumor shrinkage lead to symptomatic relief [126]. In a phase 2 study involving 532 patients with GCTB who received denosumab and were followed-up for a median of 58 months, patients developed grade 3 or severe adverse events, such as hypophosphatemia (5%), osteonecrosis of the jaw (3%), pain in the extremities (2%), anemia (2%), atypical femur fracture (1%), and hypercalcemia (1%) [127]. A dosing interval of 3 months is recommended to reduce these complications [15,128]. Pregnancy is an absolute contraindication to denosumab therapy, while the long-term effects of denosumab treatment on child delivery are unknown [129]. Given that GCTB is generally diagnosed in women of childbearing age, this important point should be investigated in detail in the future. Recently, Chandler et al. [130] reported secukinumab (anti-IL-17 antibody) treatment for psoriatic arthritis with significant intralesional calcifications in a patient with GCTB. Histological examination revealed ossification, new bone formation, and remodeling [130]. A paucity of osteoclast-type giant cells has also been reported [130]. Because secukinumab has much milder adverse effects, including nasopharyngitis, headache, nausea, diarrhea, and pyrexia, and is not contraindicated in pregnant patients [131,132], it may be an alternative treatment to denosumab.

## 8. Malignant Giant Cell Tumor of Bone

**Figure 3:**
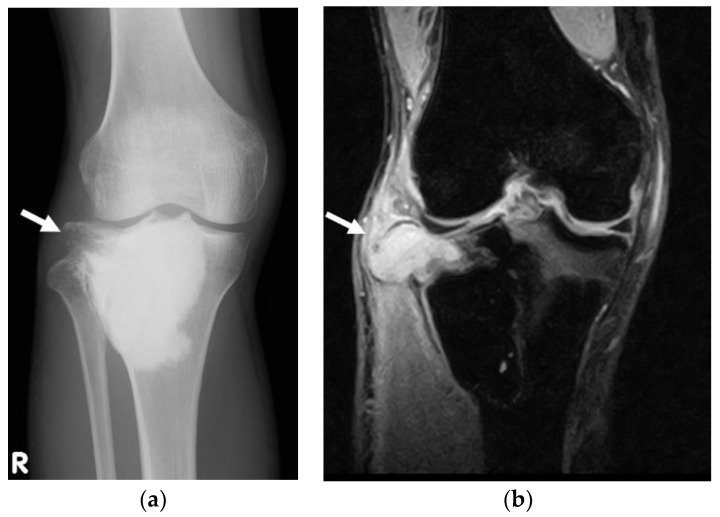
A patient with secondary malignant giant cell tumor of the proximal tibia who experienced malignant transformation after the third episode of local recurrence 6 years after the initial inversion recovery. (**a**) Radiograph shows a lytic lesion at lateral side of the proximal tibia (arrow). (**b**) The coronal fat-suppressed T2 W magnetic resonance image shows a high-intensity extraosseous lesion corresponding to a Campanacci stage 3 tumor (arrow).

Malignant GCTB can be considered either primary or secondary, accounting for 4% of all GCTBs [10,133]. Primary malignant GCTB accounts for 1.6% of all GCTBs, while secondary malignant GCTB accounts for 2.4% of all GCTBs [10]. Primary malignant GCTB is confirmed when sarcoma is diagnosed concurrently with an initial diagnosis of GCTB, while secondary malignant GCTB is diagnosed when malignancy is detected at the site of GCTB previously treated with surgery, radiotherapy, or denosumab [133]. Radiotherapy may induce late malignant transformation of GCTB [78,134,135,136,137]. The sites of both primary and secondary malignant GCTBs are similar to the distribution of benign GCTBs and are often around the knee [133]. Both primary and secondary malignant GCTB frequently occur in the 30-year age group and are slightly more common in men than in women [133]. The pathological diagnosis of primary malignant GCTB is difficult. Liu et al. reported that nine of 12 patients with primary malignant GCTB underwent needle biopsy prior to surgery, while eight (89%) of these patients were diagnosed with benign GCTB [133]. Imaging findings of primary malignant GCTB showed Campanacci stage 2 tumors in seven patients and Campanacci stage 3 in five patients [133]. Domovitov et al. investigated the prognosis of 25 patients with primary malignant GCTB over a median follow-up period of 104 months and reported that the mortality rate of primary malignant GCTB was 16% (4 of 25 patients) [138]. In contrast, the mortality rate of secondary malignant GCTB was reported to be 63% (12 of 19 patients) [139]. Liu et al. reported that 10 of 20 patients with secondary malignant GCTB were diagnosed with secondary malignant GCTB after curettage without biopsy, while the other 10 patients were diagnosed with secondary malignant GCTB after biopsy [133]. It is difficult to suspect secondary malignant GCTB based on imaging findings alone [133]; however, 19 (95%) of 20 patients with secondary malignant GCTB had Campancci stage 3 tumors (Figure 3) [133]. Late local recurrence was more frequently associated with the diagnosis of secondary malignant GCTB than early local recurrence, which is usually related to benign GCTB (median: 57 months vs. 19 months), with a cutoff time of 4 years being reported [133]. GCTBs that metastasize to the lungs should be suspected for malignant transformation [133]. In addition, cases with a malignant transformation time of <1 year were possibly cases of primary malignant GCTBs [133]. As local recurrence of benign GCTB usually occurs within 2 years of surgery [29], malignant transformation should be suspected when the interval between the last surgery and local recurrence is >2 years (“late” local recurrence).

According to recent systematic reviews, the cumulative incidence of secondary malignant GCTB without a history of radiotherapy or denosumab treatment was 0.6% [10]. In the literature, 18 cases of malignant transformation of GCTB during and after denosumab treatment have been reported [12,13,16,20,21,22,23,24,25,26,27]. Chawla et al. followed-up 526 patients with GCTB for a median follow-up of 58 months after denosumab administration and reported that malignant transformation occurred in four (0.8%) patients [127]. In these four patients, the time from diagnosis of GCTB to malignant transformation ranged from 17 months to 11 years [140]. Chawla et al. reported that the incidence of confirmed malignant transformation in patients treated with denosumab in their study was similar to that in patients who did not receive denosumab treatment in previous studies [127]. Agarwal et al. reported that at a median follow-up of 27 months after denosumab administration to 25 patients with GCTB, malignant transformation occurred in one (4%) patient 8 months postoperatively [16]. Treffel et al. reported that among 35 patients who had GCTB and received denosumab treatment, malignant transformation occurred in one (2.9%) patient 18 months postoperatively [27]. Recently, Perrin et al. reported that at a median follow-up of 57 months after denosumab administration to 25 patients with GCTB, malignant transformation occurred in one (4%) patient 55 months postoperatively [25]. Longer follow-up of patients treated with denosumab is needed to confirm the safety of denosumab treatment for GCTB.

Wide resection and (neo-)adjuvant chemotherapy are recommended for localized malignant GCTB [133,141]. Anract et al. [141] reported better 1-year survival rates in patients who underwent surgery with adjuvant chemotherapy than in those who underwent surgery alone; however, the 5-year survival rates did not improve. This study also reported that in three of four patients who had malignant GCTB and received neoadjuvant chemotherapy, the resected specimen showed tumor response [140]. Liu et al. [133] reported that the overall survival rates of patients treated with adjuvant chemotherapy did not improve, although adjuvant chemotherapy was beneficial for lung-metastasis-free survival. The 5-year survival rates in the chemotherapy and non-chemotherapy groups were 57.0% and 33.3%, respectively (*p* = 0.167) [133]. The median pulmonary-metastasis-free survival rate was significantly longer in patients who received chemotherapy than in those who underwent surgery alone (13 months vs. 6 months) [133].

## 9. Future Direction

Randomized control trials comparing malignant transformation rates between denosumab-treated and non-denosumab-treated groups are needed to prove the safety of denosumab. This trial will require a follow-up period of 10 years or more. Zolendronic acid is much less expensive than denosumab and seems to have a lower risk of inducing malignant transformation than denosumab [142]; therefore, prospective comparative studies of the efficacy of denosumab and zorendronate for GCTB should be performed prior to en bloc resection and in patients with inoperable lesions or lung metastases. Furthermore, it is necessary to develop a drug that has fewer side effects than denosumab, can be used in pregnant women, and has the same effect as denosumab. In addition, to clarify the effect of adjuvant chemotherapy for localized malignant GCTB, a randomized control trial comparing the surgery and adjuvant chemotherapy and surgery alone groups is needed in the future.

## 10. Conclusions

According to the World Health Organization classification in 2020, GCTB was corrected from a benign to an intermediate malignancy. The H3F3A mutation was found to be a specific marker for GCTB and became essential for the diagnosis of GCTB. Curettage is the standard treatment for GCTB, and preoperative denosumab treatment should be avoided, as it increases the risk of local recurrence. Curettage is an optional treatment for local recurrence to achieve good functional outcomes. The presence of pathological fractures is not a contraindication for curettage. Radiotherapy is associated with a risk of malignant transformation and should be limited to unresectable lesions, such as the spine and pelvis, where denosumab, zoledronic acid, or embolization is not available. Denosumab should be administered preoperatively along with en bloc resection for GCTB in the pelvis and spine, as it is easier to perform surgery after denosumab therapy. Nerve-sparing surgery after embolization could be an option for GCTB of the sacrum. Denosumab therapy with or without embolization should be indicated for inoperable GCTB of the pelvis, spine, and sacrum. It is recommended to adopt the wait-and-see approach for lung metastases and then administer denosumab to control the growing lesions. Late local recurrence should be suspected as a malignant transformation.

## Figures and Tables

**Table 1:** Scoring system for giant cell tumors of the bone around the knee proposed by Lun et al. [66].

Parameters	Score
Pathological fracture	
No fracture	0
Simple fracture	2
Complicated fracture	3
Cortical bone destruction	
No destruction	0
Thinned but intact bone cortex	2
Penetration of bone cortex	3
Tumor size	
Small size (<50 mm^3^)	1
Moderate size (50–100 mm^3^)	2
Large size (>100 mm^3^)	3
Damaged subchondral bone and articular cartilage involvement	
Subchondral bone damaged	1
Articular cartilage involvement without penetration	2
Penetration of articular cartilage	3

**Table 2:** Classification for giant cell tumors using the scoring system and surgical decision making proposed by Lun et al. [66].

Total Scores	Surgical Decision Making
2–4	Intralesional curettage
5–9	Intralesional curettage with internal fixation
10–12	En bloc resection and reconstruction with a prosthesis or allograft
